# Reciprocal Regulation of Cancer-Associated Fibroblasts and Tumor Microenvironment in Gastrointestinal Cancer: Implications for Cancer Dormancy

**DOI:** 10.3390/cancers15092513

**Published:** 2023-04-27

**Authors:** Shih-Hsuan Cheng, Hsin-Ying Clair Chiou, Jiunn-Wei Wang, Ming-Hong Lin

**Affiliations:** 1 Department of Microbiology and Immunology, School of Medicine, College of Medicine, Kaohsiung Medical University, Kaohsiung 807, Taiwan; aibon03@hotmail.com; 2 Division of Gastroenterology, Department of Internal Medicine, Kaohsiung Medical University Hospital, Kaohsiung Medical University, Kaohsiung 807, Taiwan; 3 Teaching and Research Center, Kaohsiung Municipal Siaogang Hospital, Kaohsiung Medical University, Kaohsiung 812, Taiwan; 4 Kaohsiung Medical University Hospital, Kaohsiung Medical University, Kaohsiung 807, Taiwan; 5 Department of Internal Medicine, School of Medicine, College of Medicine, Kaohsiung Medical University, Kaohsiung 807, Taiwan; 6 Graduate Institute of Clinical Medicine, College of Medicine, Kaohsiung Medical University, Kaohsiung 807, Taiwan; 7 Department of Medical Research, Kaohsiung Medical University Hospital, Kaohsiung Medical University, Kaohsiung 807, Taiwan; 8 Department of Post Baccalaureate Medicine, College of Medicine, Kaohsiung Medical University, Kaohsiung City 807, Taiwan; 9 Graduate Institute of Medicine, College of Medicine, Kaohsiung Medical University, Kaohsiung 807, Taiwan; 10 Master of Science Program in Tropical Medicine, College of Medicine, Kaohsiung Medical University, Kaohsiung 807, Taiwan

**Keywords:** the tumor microenvironment (TME), cancer-associated fibroblasts (CAFs), cytokines, dormancy

## Abstract

**Simple Summary:**

Gastrointestinal (GI) cancers are the leading cancer-related deaths worldwide. Despite improved survival rates with current treatments, recurrence remains a clinical challenge for patients with GI cancers. The unmet gap still needs to be further investigated. The tumor microenvironment (TME) is associated with the dormancy and awakening of tumor cells. Among them, the reciprocal regulation of cytokines/chemokines between cancer-associated fibroblasts (CAFs) and TME is a key driver of tumor progression, metastasis, and resistance to chemotherapy. In this review, we discuss the involvement of cytokines/chemokines produced by CAFs in the development of resistance to gastrointestinal cancer therapy, including tumor dormancy and tumor regeneration. Deciphering the crosstalk of CAFs and TME thus reduces the therapeutic recurrence of GI cancers.

**Abstract:**

Gastrointestinal (GI) cancers remain a major cause of cancer-related deaths worldwide. Despite the progress made in current treatments, patients with GI cancers still have high recurrence rates after initial treatment. Cancer dormancy, which involves the entry and escape of cancer cells from dormancy, is linked to treatment resistance, metastasis, and disease relapse. Recently, the role of the tumor microenvironment (TME) in disease progression and treatment has received increasing attention. The crosstalk between cancer-associated fibroblasts (CAF)-secreted cytokines/chemokines and other TME components, for example, extracellular matrix remodeling and immunomodulatory functions, play crucial roles in tumorigenesis. While there is limited direct evidence of a relationship between CAFs and cancer cell dormancy, this review explores the potential of CAF-secreted cytokines/chemokines to either promote cancer cell dormancy or awaken dormant cancer cells under different conditions, and the therapeutic strategies that may be applicable. By understanding the interactions between cytokines/chemokines released by CAFs and the TME, and their impact on the entry/escape of cancer dormancy, researchers may develop new strategies to reduce the risk of therapeutic relapse in patients with GI cancers.

## 1. Introduction

Gastrointestinal (GI) cancer is a malignant disease that affects the gastrointestinal system, including the esophagus, liver, stomach, gallbladder and bile ducts, pancreas, gastrointestinal interstitial tumor (GIST), and colon. Despite advances in the treatment of GI cancers, they still account for 35% of all cancer-related deaths, posing a major threat to public health [1]. One of the most pressing challenges in treating GI cancers is the resistance of cancer cells to therapy.

The tumor microenvironment (TME) is a complex environment consisting of immune cells, cancer-associated fibroblasts (CAFs), the extracellular matrix (ECM), cytokines, chemokines, and growth factors, which play important roles in tumorigenesis, metastasis, and cancer cell resistance to therapy [2]. From among them, CAFs are the essential components of the TME in GI cancers as they can trigger the resistance of cancer cells to treatment [3].

Cancer recurrence can occur shortly after treatment or several years after the initial diagnosis or treatment, indicating that the cancer cells have been reactivated and are proliferating (also known as awakening) after a long period of latency (also known as dormancy) [4]. Cancer cell dormancy is an adaptive strategy that allows cancer cells to survive in disadvantageous conditions that they must deal with [5]. Dormant cancer cells (DCCs) may reactivate due to the crosstalk between cytokines/chemokines secreted by CAFs and other components of the TME, including the remodeling of the ECM and immunomodulatory functions.

Patients with gastrointestinal (GI) cancers have been experiencing shorter recurrence periods compared to certain other cancer types, such as breast cancer [6,7,8,9], mainly because of the insidious nature of their symptoms and the absence of effective early detection screening. Consequently, many cases are diagnosed at advanced stages, which results in fewer treatment options and a higher probability of developing drug resistance, with the alterations in the TME being a critical factor that contributes to the development of resistance in GI cancers [10,11]. In this review, we discuss the role of cytokines/chemokines secreted by CAFs in the TME of GI cancers and the generation of therapeutic resistance through the mechanism of cancer dormancy. Notably, the reciprocal regulation between CAF-derived cytokines/chemokines and the TME is not unique to GI tumors, and the underlying mechanisms may differ from those in other solid tumors due to the high heterogeneity of CAFs. Deshka et al. showed that CAF characteristics vary across solid tumor types and species. Nevertheless, CAFs can be classified into three superclusters: steady-state-like (SSL), mechanoresponsive (MR), and immunomodulatory (IM), using paired, same-cell chromatin accessibility and transcriptome analysis [12]. Additionally, Galbo et al. found that different CAF isoforms may activate their own unique molecular pathways to regulate the tumor microenvironment in various cancer types [13].

As direct evidence of the relationship between CAFs and cancer cell dormancy is limited, we have summarized the potential of cytokines/chemokines secreted by CAFs to induce or awaken DCCs under different conditions and the agents that may be applicable to resist it. Deciphering the crosstalk between CAFs and the TME in relation to the mechanisms of cancer cell dormancy may provide insightful strategies to further prevent and treat GI cancer recurrence. Our review aims to provide a better understanding of the role of CAFs in GI cancers, and to highlight potential therapeutic targets that could reduce the risk of recurrence in patients with GI cancers. 

## 2. Reciprocal Regulation of CAF-Secreted Cytokines/Chemokines and the Gastrointestinal Tumor Microenvironment

**Figure 1:**
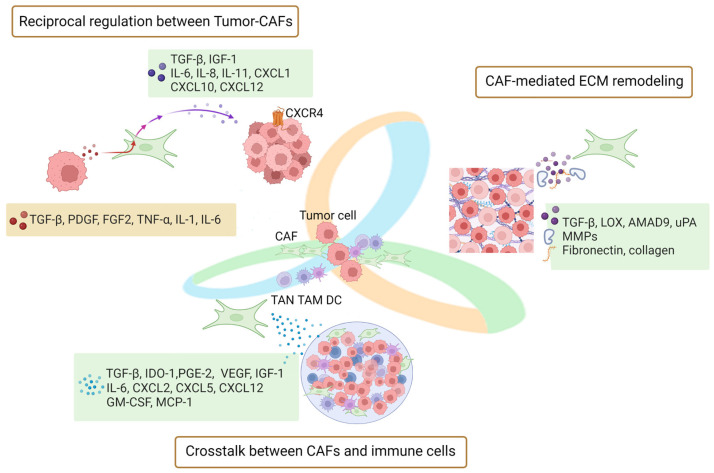
Reciprocal regulation of CAF-secreted cytokines/chemokines and the tumor microenvironment (TME). The crosstalk between CAFs and TME contributes to tumor progression through three main mechanisms: (1) The behavior of CAFs can be influenced by cytokines produced by tumor cells. In turn, these cytokines stimulate CAFs to secrete pro-tumorigenic factors, which contribute to the tumorigenic process. (2) By producing and cross-linking ECM components, CAFs regulate the structure and function of the ECM to support tumor progression and metastasis. (3) CAF-derived factors play a regulatory role in immune cells, including tumor-associated macrophages (TAMs), tumor-associated neutrophils (TANs), and dendritic cells (DCs). Together, these factors establish a tumor immune microenvironment (TIME) that is conducive to tumor growth.

The GI TME is a dynamic environment, dominated by CAFs through direct contact and indirect paracrine effects on the tumor–stroma crosstalk, ECM remodeling, and immune cells [14,15]. The multi-regulatory functions of cytokines/chemokines secreted by CAFs in the TME are depicted in Figure 1.

### 2.1. Crosstalk between Tumor and CAFs Contributes to Tumor Progression

Tumor cells set the conditions in the microenvironment and then take advantage of it, while CAFs maintain the microenvironment and further promote tumorigenesis [16]. The transforming growth factor beta (TGF-β) is a cytokine required for the induction of fibrotic responses and the activation of the tumor stroma, and it subsequently induces the formation of CAF phenotypes in the TME [17,18]. After fibroblast activation, TGF-β is secreted by CAFs and facilitates the further development of tumor cells [19]. Similarly, other factors released by tumor cells, such as the platelet-derived growth factor (PDGF) and fibroblast growth factor 2 (FGF-2), or inflammatory signals such as interleukin (IL)-1, IL-6 and tumor necrosis factor alpha (TNF-α), recruit and proliferate additional CAFs. Therefore, the activated CAFs shape a pro-tumorigenic TME through a variety of functions, which facilitates tumor progression [20].

Moreover, CXC ligand 12 (CXCL12, also called stromal cell-derived factor-1, SDF-1), which is produced by stromal cells and binds to C-X-C chemokine receptor type 4 (CXCR4) on the surface of tumor cells, has been reported to be essential for cancer pathogenesis. In gastric cancer (GC), the high expression of CXCL12 by CAFs may influence the malignancy of tumor cells [21]. More specifically, in a study of pancreatic cancer, tumor cells harboring the mutant KRAS^G12D^ communicated with CAFs via sonic hedgehog signaling, which stimulated the production of insulin-like growth factor 1 (IGF-1) in CAFs; in turn, reciprocal signaling affected tumor cell proliferation [22].

Recent studies have reported that CAFs can sense tumor cell genomic stress by expressing interferon (IFN)-β1, which leads to the increased production of associated factors such as CXC ligand 1 (CXCL1) and CXC ligand 10 (CXCL10), IL-6, IL-8, and IL-11 [23]. Among them, IL-6 and IL-8 are considered as indicators and therapeutic targets in GI-related cancers [24,25,26,27]. For example, CAF-secreted IL-6 enhances tumor cells’ epithelial–mesenchymal transition (EMT) via the activation of the Janus kinase 2/signal transducers and activators of the transcription 3 (JAK2/STAT3) pathway in GC [28]. Activation of STAT3 by CAF-derived IL-6/IL-11 promotes the development of colorectal tumors and is associated with a poor prognosis [29,30]. Furthermore, a meta-analysis including 12 studies showed that high IL-8 levels were significantly associated with shorter overall survival and progression-free survival in colorectal cancer (CRC) patients [26]. In summary, there is a bidirectional crosstalk between CAFs and tumor cells that shapes a dynamic TME through the release of cytokines/chemokines, thus aggravating the progression of GI cancers.

### 2.2. CAF-Mediated ECM Remodeling in Tumor Progression

The dysregulation of ECM homeostasis is associated with cancer progression and drug resistance [31]. CAF-derived cytokines/chemokines have an impact on ECM structure and function, and all of them are involved in tumor-promoting and metastasis-accelerating ECM remodeling. CAF-derived TGF-β upregulates matrix metalloproteinase 9 (MMP-9), which remodels ECM components and creates a favorable environment for tumor progression and metastasis [32]. Another enzyme regulated by TGF-β is lysyl oxidase (LOX), which cross-links collagen and elastin fibers. This leads to ECM stiffness and further feedback to TGF-β induction and contributes to a tumor-promoting inflammatory environment [33,34]. CAFs also secrete a disintegrin and metalloproteinase 9 (ADAM9), which has been implicated in increasing the proliferation and dissemination of CRC cells [35]. Recent literature suggests that ADAMs are considered biochemical markers of GI tumorigenesis due to their protein hydrolysis and adhesion activities [36,37]. Overall, CAF-derived cytokines/chemokines play a significant role in ECM remodeling, further contributing to tumor progression and metastasis.

### 2.3. Crosstalk between CAF-Derived Factors and Immune Cells in the TME

CAF-derived cytokines/chemokines play a critical role in modulating the immune response and are associated with immunosuppressive TME [38].

CAFs secrete various cytokines/chemokines that promote the recruitment and polarization of immune inhibitory cells, such as tumor-associated macrophages (TAMs), tumor-associated neutrophils (TANs), and regulatory T cells. For example, monocyte chemotactic protein-1 (MCP-1), IL-6, CXCL12, and granulocyte-macrophage colony-stimulating factor (GM-CSF) promote the recruitment and polarization of these immune inhibitory cells [39,40,41]. In addition, CAFs secrete CXCL2 [42] and CXCL5 [43] to promote the PD-L1 expression of cancer cells via phosphatidylinositol-3-kinase (PI3K)/AKT signaling, which further facilitates immune deviation toward tolerance.

Moreover, CAFs also secrete TGF-β, indoleamine 2,3-dioxygenase (IDO), and prostaglandin E2 (PGE-2), which interfere with the activation of natural killer cells and thus suppress innate anti-tumor immunity [44,45]. CAFs secrete IL-6, CXCL12, and vascular endothelial growth factor (VEGF), which combat the activity of cytotoxic T lymphocytes and the maturation of dendritic cells (DCs) [46].

Understanding the mechanisms of CAF-induced immunosuppression and their interactions with immune cells within the TME can aid in identifying potential molecular targets for CAF-targeted therapy. Further exploration of these interactions may offer new therapeutic strategies to enhance the anti-tumor immune response in the TME.

## 3. CAF-Secreted Cytokines/Chemokines Inducing Cancer Cell Dormancy

**Figure 2:**
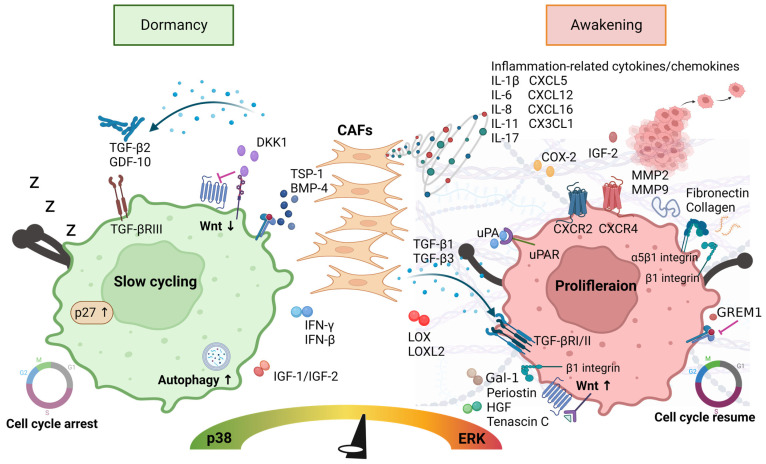
The ratio of p38 signaling to ERK determines whether cancer cells enter a dormant state or undergo proliferation. Various CAF-secreted factors can induce cancer cells to enter a dormant state, which is characterized by cell cycle arrest through upregulation of p21, as well as increased autophagy and inhibition of Wnt signaling. On the other hand, CAFs can secrete factors to promote the awakening of dormant cancer cells by restoring the cell cycle and promoting the Wnt signaling pathway. In addition, CAFs can promote the escape of dormant cancer cells through the synthesis and remodeling of the ECM. These processes promote cytoskeletal reorganization, β1 and α5β1 integrin signaling, stromal stiffness, and the adhesion of tumor cells to the ECM, ultimately leading to dormant cell escape. Some CAF-secreted factors, such as IL-6 and IGF-2, play a dual role.

Cancer cell dormancy refers to the state in which cancer cells survive through slow proliferation or slow cycling [47]. DCCs themselves activate mechanisms such as autophagy or immune evasion, which is the result of their interaction with the TME. In other words, dormancy can be considered an adaptive response to microenvironmental stress [48], where CAF-secreted cytokines/chemokines may influence DCCs directly or indirectly by interaction with the TME (Figure 2). The activation of the extracellular signal-regulated kinase (ERK) pathway has been demonstrated to facilitate G0-G1-S-phase transition and promote cell division. In contrast, high levels of p38 activity have been implicated as negative regulators of growth by inhibiting ERK, inducing G0/G1 arrest, or triggering apoptosis. As a result, the ratio between p38 signaling and the ERK determines whether tumor cells proliferate or enter a dormant state [49,50].

CAFs are one of the sources of TGF-β2, which has been implicated in the maintenance of cancer cell dormancy [51]. TGF-β2 induces SMAD1/2/5 to upregulate p27 via TGFβ-RIII, which in turn activates p38 to promote dormancy [52]. In addition, Wu et al. reported that TGF-β2 signaling decreased the proliferation of both CAFs and tumor cells by activating the G1/S checkpoint in CRC [53]. In addition, an elevated production of TGF-β2 by CAFs was observed when heat shock factor (HSF)-1 was activated [54]. HSF-1 is a transcription factor that responds to a state of cellular hypoxia and is associated with dormancy through the induction of cell cycle arrest and the maintenance of cell survival through the activation of autophagy [55,56]. Moreover, CAFs secrete growth differentiation factor 10 (GDF-10) [57], which promotes cell cycle arrest by binding to the TGF-βRIII receptor and then activating the p38MAPK signaling pathway [58]. Furthermore, Shirai et al. found that bone morphogenetic protein 4 (BMP-4) induced G1 arrest in diffuse gastric cancer cells via SMAD-induced p21 [59]. Similarly, Yokoyama et al. reported that the autocrine signaling of BMP-4 may be a therapeutic target in CRC [60]. In summary, in the GI cancer TME, those CAF-secreted factors mentioned above promote or maintain cancer cell dormancy by upregulating the p38 pathway and/or inducing cell cycle arrest.

Recently, Owen et al. revealed a key role of intrinsic interferon (IFN) signaling in maintaining the dormant state of tumor cells [61]. IFN-β secreted by CAFs drives the sustained activation of the type I interferon receptor/interferon regulatory factor 7 pathway, triggering the chemotherapy-induced dormancy of breast cancer cells [62]. In line with this, Guo et al. suggested that IRF7 is associated with immune cell infiltration in gastric adenocarcinoma, which serves as a prognostic biomarker [63]. Therefore, IFN-β may play a role in inducing cancer cell dormancy in the GI microenvironment. Additionally, CAFs can produce IFN-γ, which activates STAT1 and thus induces cells to enter G0/G1 growth arrest by reducing the cyclin D1/CDK4 complex [64]. On the other hand, IFN-γ can mediate cancer cell dormancy via the indoleamine-2,3-dioxygenase/kynurenine/aryl hydrocarbon receptor (AhR)/p27 pathway in a STAT1-independent manner [65]. Taken together, it is suggested that CAF-secreted IFN-γ is critical for the induction of cancer cell dormancy.

The Wnt pathway, an inducer of proliferation, is involved in the reactivation of DCCs. Dickkopf-related protein 1 (DKK-1), which is secreted by CAFs, is an inhibitor of Wnt signaling, and its expression has been reported to be a prognostic factor in the prediction of tumor recurrence and survival in patients with advanced GC [66]. Malladi et al. found that isolated latency-competent cancer cells from early-stage human lung and breast cancers enter a dormant state by expressing a Sox-dependent stemness state and the Wnt inhibitor DKK1 [67]. 

Another feature of DCCs is the activation of autophagy in response to different forms of stress and the promotion of cancer cell survival during growth arrest, eventually making autophagy integral to the survival of DCCs. Therefore, it is essential to maintain autophagic flux in cancer cells that are in a dormant state [68,69]. In both in vitro and in vivo research models, CAFs produce IGF-1/IGF-2 to induce autophagic dormancy, promoting cancer cell recovery from radiation-induced damage [70] and protecting cells from chemotherapeutic stress. In addition, Rajbhandari et al. reported that autocrine IGF-1 signaling mediated the dormancy of pancreatic tumor cells in the absence of oncogenic drivers [71]. Lu et al. reported that IL-6 induces autophagy through the expression of NS5ATP9, which upregulates IL-6 levels and further induces autophagy [72]. 

Finally, the balance of pro- and anti-angiogenic factors produced by CAFs, such as VEGF for the former and thrombospondin-1 (TSP-1) for the latter, is also involved in the regulation of cancer cell dormancy [73]. The loss of TSP-1 has been shown to promote escape from cancer dormancy [74,75], in which higher levels of TSP-1 in the lesion tissues of patients with metastatic CRC have been reported to result in the remission of mortality [76].

## 4. CAF-Secreted Cytokines/Chemokines Awakening Cancer Cell Dormancy

Dormancy escape can be stimulated by evolving interactions with TME components, with CAF-driven cytokines/chemokines being one of the main driving forces [47]. Awakening is the process of forcing DCCs back into the cell cycle, shifting the imbalance from a non-proliferating state of p38 MAPK induction to a proliferating state of ERK upregulation [77]. 

The CAF-secreted urokinase-type plasminogen activator (uPA) binds to its receptor (uPAR), activating the integrin and epidermal growth factor receptor (EGFR) pathways and resulting in the subsequent activation of ERK and reduction of p38 activity, which promotes tumor cell proliferation [78]. Likewise, the inhibition of uPAR-activated FAK signaling leads to low levels of ERK and induces cancer cell dormancy. Taken together, the uPA/uPAR axis is critical in the awakening of DCCs [79]. 

In addition to the ERK pathway, the previously mentioned Wnt signaling is an indicator of cell proliferation, and it also reactivates DCCs [80]. Tenascin C is known to be initially produced by initiated metastatic tumor cells and later secreted by CAFs to support the growth of metastatic cells by promoting the Wnt signaling pathway; this appears to reflect the awakening of DCCs [81]. In this regard, the knockdown of Tenascin C inhibits ERK signaling and thereby inhibits the metastasis of gastric cancer. Furthermore, studies have reported that CAFs secrete periostin and hepatocyte growth factor (HGF), which promote the recruitment of Wnt ligands and thus increase Wnt presentation in cancer cells, including colon and gastric cancer [82,83,84], suggesting that the reawakening and metastasis of dormant cancer cells are likewise promoted [85]. Many studies on gastrointestinal cancers have shown that the upregulation of periostin and HGF is associated with increasing cancer cell malignancy, the elevating of metastatic capacity, and worsening patient prognosis [86,87,88]; thus, it can be said that both periostin and HGF allow dormant cancer cells to reawaken and metastasize. In addition, galectin-1 (Gal-1) secreted by CAFs binds to integrin β1, leading to the activation of the Wnt/β-catenin signaling pathway associated with the metastatic process in GC cells [89,90]. 

Chronic inflammation is known to promote the development and progression of cancer [91] and is now also considered to be a major driver in the awakening of dormant cancer cells [92]. During chronic inflammation, the TME is enriched with inflammatory factors secreted by tumor cells, CAFs, and immune cells, such as IL-6, IL-8, IL-1β, IL-10, and IL-17, creating a microenvironment conducive to dormancy escape as well as reactivating cancer cell expansion [92]. For example, Khazali et al. demonstrated that IL-8 can mediate cancer cell dormancy escape using an in vitro 3D liver microphysiological system [93]. Furthermore, IL-17 secreted by CAFs upregulates JAK2/STAT3 signaling, which promotes gastric cancer cell malignancy [94]. Similarly, IL-17 has been shown to promote the transformation of dormant gastric cancer stem cells [95]. Cyclooxygenase 2 (COX-2) is a pro-inflammatory mediator and a key mediator of inflammatory pathways that awaken DCCs [96,97]. COX-2 is also abundant in the TME of GI cancers [98], where CAFs are a major source of COX-2 [99].

Regarding IL-6, as described in the article above, it is secreted by CAFs, which promote cancer cell dormancy by inducing autophagy. Nevertheless, most studies have concluded that IL-6 is a key player in the inflammatory process, serving as a pro-inflammatory and pro-metastatic factor and thus promoting the escape of DCCs [100]. For example, a study by Shaashua et al. showed that spontaneous micrometastatic cells returned to a dormant state after IL-6 was removed during a minimally invasive resectioning of the primary tumor, highlighting the role of IL-6 in dormancy escape [101]. Furthermore, Esposito et al. showed that IL-6 disrupts the dormant phenotype mediated by the tumor suppressor aplasia Ras homolog member (ARH-I) by upregulating an onco-miRNA miR-1305 [102]. In sum, IL-6 plays a role in controlling cancer cell dormancy or awakening, depending on the interaction between the stage of the tumor cell progression and the TME.

CAFs secrete chemokines, which act as mediators of inflammation and influence the entry/escape of dormancy. As mentioned earlier, the interaction between immune and tumor cells in the TME has important implications for tumor development and treatment, and the chemokines secreted by CAFs act as activators for this [34]. Romero-Moreno et al. used in vitro co-culture to simulate the final stage of metastasis of dormant breast cancer cells to the bone. They found that the increase in CXCL5 at the metastatic site created a favorable and supportive microenvironment that promoted the reactivation and recolonization of DCCs [103]. Similarly, CXCL5 and its receptor, CXCR2, have been shown to be associated with metastasis in many gastrointestinal cancer models [104,105] and may be potential therapeutic targets for TME [106,107]. Adamski et al. showed that CXCL12, CXCL16, and CX3CL1 result in the delayed entry and exit of GBM cells from temozolomide-induced cellular dormancy. In other words, CXCL12, CXCL16, and CX3CL1 facilitate the escape of cells from the dormant state [108]. Furthermore, there is evidence that CCXR4 is absent or low in dormant gastric cancer cells [95] or metastatic breast cancer cells [109]. On the contrary, the increased expression of CXCL12, a ligand for CXCR4, facilitates the escape of cancer cells from dormancy. Similarly, Peng et al. showed that the activation of TGF-β via integrin αvβ6 further stimulates CXCL12 secretion by CAFs, leading to CRC invasion via the CXCL12/CXCR4-dependent axis [110].

Notably, some CAFs release factors that play opposite roles in the TME, either by mediating dormancy or by reversing it; in addition to IL-6, these also include TGF-β. In contrast to the ability of TGF-β2 to promote dormancy, TGF-β1 has been shown in several studies to be associated with tumor cell proliferation [111] and the awakening of dormant tumor cells [112]. For example, in sprouting neovascularization, TGF-β1 blocked the quiescent effects of TSP-1 and promoted micrometastasis [112]. Interestingly, the TGF-β1 ligand antagonist Coco was shown to reactivate dormant breast cancer cells in the lung, but not in the bone or brain, suggesting the organ-specific regulation of cancer cell dormancy [113]. Similarly, CAF-secreted IGF-2 has a dual role in dormancy entry/escape. In contrast to the ability of IGF-2 to promote dormancy through the induction of autophagy, CAFs produce high levels of IGF-2, which is thought to promote distant metastasis by stimulating the formation of a pre-metastatic ecotone [114]. Furthermore, in an esophageal cancer model, tumor-derived IGF-2 induces CAFs to produce VEGF, creating a general environment for metastasis by stimulating the formation of a pre-metastatic ecotone [115]. It was thus concluded that IGF-2 plays a role in dormancy escape.

Another dominant ability of CAFs to drive dormancy escape is their ability to synthesize and remodel ECM, further affecting the balance between ECM-mediated cell dormancy and proliferation [116]. For example, CAFs assemble the ECM protein fibronectin (FN), which promotes dormancy escape by inhibiting the p38 signaling pathway and activating ERK [117,118]. In detail, CAFs secrete MMP-2, MMP-9, and type I collagen, which are important processes that promote cellular dormancy escape by inducing cytoskeletal reorganization and β1/β4 integrin signaling [52,119,120]. In addition, CAFs secrete LOX and lysyl oxidase-like (LOXL2), which are involved in collagen deposition. This determines the matrix stiffness and influences tumor cell adhesion to the ECM, thus allowing the transition from a dormant to a proliferative state [121,122,123]. Moreover, CAF-secreted Gremlin 1 (GREM1), a BMP antagonist, was found to be upregulated in GI cancers [124]. GREM1 has also been detected in the demyelinating invasion front of CRC, suggesting a potential role in cancer metastasis [124,125]. In addition, GREM1 enables dormant breast cancer cells to escape the quiescence imposed by BMP signaling and thus initiate metastasis [113].

Furthermore, other cell types in TME are involved in ECM remodeling. For example, Albrengues et al. discovered that, in an inflammatory state, neutrophil elastase and MMP-9, two neutrophil extracellular trap (NET) proteases, can sequentially cleave laminin (LN), which can result in the activation of integrin α3β1 signaling and the proliferation of dormant cancer cells. The proteolytic remodeling of LN can be prevented by blocking NET remodeling with antibodies, which can prevent the awakening of dormant cells [120]. Moreover, MMPs can disrupt blood vessels in metastatic tissues, leading to the production of TGF-β1 and ECM molecules, such as periostin and FN, by endothelial cells and contributing to the deposition of ECM components [112]. These findings emphasize the importance of the interaction between the TME and ECM in the development of cancer dormancy escape and metastasis. 

In addition to the previous discussion, the composition of the ECM plays an important role in chemoresistance and immunosuppression. For example, the aberrant expression of FN can activate cellular signaling pathways, such as PI3K/Akt, ERK, and FAK signaling, through interaction with tumor cell α5β1 or β1 integrins, which leads to tumor cell resistance to chemotherapeutic agents [126]. Additionally, cancer cell adhesion to the ECM can result in a drug resistance phenotype, termed cell adhesion-mediated drug resistance [127]. For instance, the activation of the LN and its receptor mGr1-Ag/37LRP pathway can reduce drug accumulation and inhibit apoptosis, resulting in GC cell resistance [128]. Moreover, collagen has been suggested as a predictor and a strategic target for the improvement of the efficacy of immune checkpoint inhibitors in anti-PD-1 responses by promoting the infiltration and accumulation of effector CD8+ T cells in tumors [129,130].

## 5. Therapeutic Strategies Targeting CAF-Secreted Cytokines/Chemokines That Maintain or Awaken Dormancy

**Figure 3:**
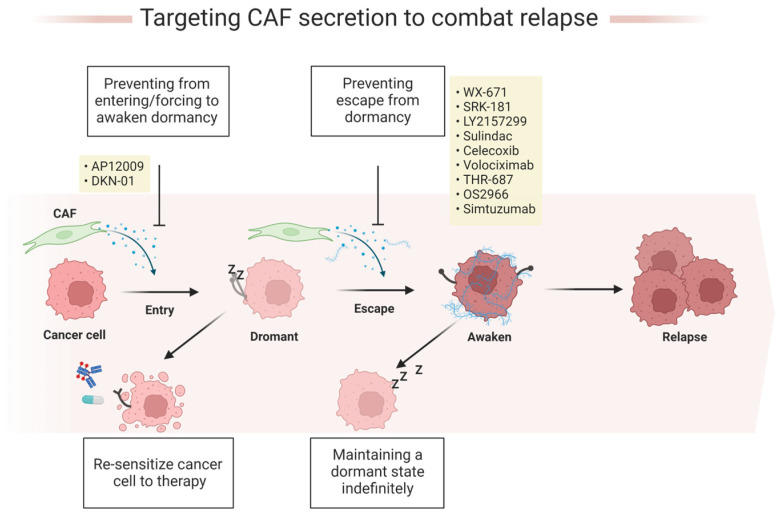
Strategies to fight cancer recurrence. Targeting dormant cancer cells is a promising approach for preventing cancer recurrence. (1) Awakening dormant cancer cells and making them sensitive to treatment. This can be achieved by either blocking the secretion of dormant drivers by CAFs or by restoring cell proliferation signals. (2) Maintaining the permanent dormancy of cancer cells to prevent regeneration. This can be achieved by inhibiting the CAF-mediated dormancy escape pathway. By targeting these dormant cells, we can prevent cancer recurrence and improve patient outcomes.

In early studies, cancer cell dormancy had already been identified as a key driver of drug resistance and cancer relapse, yet DCC remains difficult to monitor in patients, representing a gap in the fight against drug resistance. Dormancy entry/escape is a dynamic interaction between tumor cells and the TME, and the factors secreted by CAFs play an essential role in this process. Therefore, one strategy to combat cancer relapse may be to select factors secreted by CAFs as therapeutic targets (Figure 3) [131]. Here, we focus on relevant drugs that target factors secreted by CAFs and may therefore help combat dormancy-related mechanisms, particularly in GI cancers.

### 5.1. Prevention of Dormant Cancer Cell Reawakening by Targeting Their Crosstalk with Their Supportive Microenvironment

Maintaining tumor cells in a dormant state prevents metastatic cancer from recurring. In other words, it is necessary to develop new therapeutic strategies that prevent cancer recurrence by maintaining a dormant state, which could be based on indefinitely blocking the crosstalk between CAF-driven signaling and the supportive microenvironment involved in dormancy escape [132]. 

First, uPAR may be an attractive target for maintaining the dormant state of cancer cells and thus inhibiting the metastatic process of cancer [133,134]. For example, the antibody ATN-292 reduces the migration of human pancreatic cancer cells by inhibiting the binding of uPA to uPAR [135]. Qin et al. developed a novel anti-uPAR monoclonal antibody that exerts antitumor effects in GC by blocking the uPA/uPAR interaction [136]. In addition, the small molecule uPA inhibitor, WX-671, was tested in a phase II trial in patients with locally advanced non-metastatic pancreatic cancer; however, in the study, the combination of WX-671 and gemcitabine, a nucleoside analog used as a chemotherapeutic agent, was shown to be well tolerated, but the survival outcomes of the combination group were similar to those in the gemcitabine-only group [137]. 

The abundance of TGF-β1 in the TME drives dormancy escape, while CAFs are the main source of TGF-β in the TME. Agents that inhibit TGF-β1, including those that interfere with the ligand–receptor interactions or the activity of the receptor kinase, can be used as a strategy to keep tumor cells in a dormant state. Some of these agents have been or are being clinically evaluated for the treatment of gastrointestinal cancers [138]. To cite an instance, SRK-181 was developed to specifically bind the pro-segment of TGF-β1, which effectively prevents the dissociation of the mature TGF-β1 from its pro-segment. This results in the maintenance of TGF-β1 in a latent state without impacting the activation of TGF-β2 or TGF-β3 [139]. Based on current evidence, it is postulated that SRK-181 exerts its inhibitory effects systemically, suggesting its potential to modulate TGF-β1 signaling pathways, including canonical or non-canonical pathways [140]. Preclinical data suggest that SRK-181 may overcome primary resistance to checkpoint inhibitors [139,141,142]. Moreover, LY2157299 is a small molecule inhibitor of TGF-βRI kinase, also known as Galunisertib, which demonstrated efficacy in a Phase Ib/II trial in combination with gemcitabine in patients with unresectable pancreatic cancer, with minimal increase in toxicity [143,144,145]. Another TGF-βRI inhibitor, Ki26894, also known as Kirin, has been shown to inhibit a GC tumor through the reduction of invasive and bone metastases [146]. 

CAFs secrete pro-inflammatory cytokines, most of which promote dormancy. Many anti-inflammatory therapies have been extensively studied and applied in cancer treatment. Among these, non-steroidal anti-inflammatory molecules such as sulindac [147,148,149] and celecoxib [150,151] exert their influence by inhibiting COX-2 activity, and they have shown positive responses in preclinical and clinical studies on gastrointestinal cancers. Sulindac is currently undergoing a phase III clinical trial (NCT01349881) investigating its use alone or in combination with eflornithine to reduce the three-year event rate of adenomas and second primary colorectal cancer in patients previously treated for stage 0 to III colon or rectal cancer. With regard to celecoxib, a meta-analysis study has demonstrated its potential in improving response rates to standard chemotherapy among patients diagnosed with advanced colorectal cancer. Nevertheless, it is worth noting that this improvement has not been shown to increase one-year survival rates [152]. Currently, another phase III clinical trial is underway to further evaluate the efficacy of oxaliplatin, leucovorin calcium, and fluorouracil in combination with or without celecoxib, in patients with stage III colon cancer who have undergone surgery (NCT01150045) [153]. Given their presumed anti-inflammatory properties, these drugs may also hold potential in inhibiting dormancy escape [154,155,156]. 

CAF-mediated ECM remodeling is an important factor in dormancy escape [157,158]. ECM-associated molecules, such as collagen and FN, regulate the roles of integrins in the transition from dormancy to proliferation through binding [159]. Therefore, an indirect approach that could be considered is to inhibit integrin activation. Aberrant integrin expression is common in GI cancers and is thought to be associated with cancer metastasis [160]. For example, α5β1 is upregulated in GC [161] and CRC [162]. Clinically, a phase Ib trial of the anti-α5β1 integrin antibody Volociximab with carboplatin and paclitaxel in advanced non-small cell lung cancer has shown a better prognosis [163]. THR-687, a pan-αv/α5β1 inhibitor, has been tested in clinical trials for diabetic macular edema [164] and retinal vascular disease. OS2966, a monoclonal antibody directed against the β1 integrin, has entered clinical trials for the treatment of high-grade glioma [165]. Consequently, these integrin-targeting drugs have been linked to the mechanism by which CAFs regulate cancer cell dormancy, and further research is needed to determine whether they are effective in preventing the awakening of DCCs.

In addition, the inhibition of LOX or LOXL2 may be an option for preventing the awakening of DCCs [166]. Benjamin et al. showed that Lox activity promotes chemoresistance in pancreatic cancer cells. They found that an elevated level of Lox activity results in an increase in the organization of collagen fibers, which reduces the benefit of gemcitabine [167]. Zhao et al. showed that the β-aminopropionitrile (BAPN) inhibition of LOX in GC cells reduced the expression and activity of MMP-2 and MMP-9 [168]. Similarly, Li et al. showed that the inhibition of LOX by BAPN hampered the outgrowth of GC liver metastasis [169]. Simtuzumab is a humanized monoclonal antibody to IgG4 with an anti-LOXL2 function, and it has been tested in phase II trials in combination with gemcitabine and FOLFIRI (folinic acid, fluorouracil, and irinotecan) in patients with pancreatic [170] and colorectal [171] cancers, respectively. In these clinical trials, Simtuzumab was generally well tolerated. However, it is possible that Simtuzumab only targeted extracellular LOXL2 and therefore demonstrated limited clinical benefit [172]. Recently, epigallocatechin gallate (EGCG) has been shown to inhibit LOXL2 and TGF-β1, and clinical trials have shown that EGCG reduces TGF-β1 signaling and collagen accumulation [173], potentially inhibiting dormancy escape. 

Another way to prevent dormant cells from awakening is to strengthen the dormant environment. As mentioned above, TSP-1 drives tumor cells into a dormant state [174]. Therefore, the administration of the TSP-1 mimetic peptide prosaposin generates a microenvironment that inhibits cancer metastasis [175]. Furthermore, the combination of 5-AZA with trans-retinoic acid (ATRA) restores the NR2F1-induced dormant program and induces TGF-β2, thereby inhibiting cancer metastasis by restoring the TGF-β-SMAD4 signaling [176]. As for therapeutic approaches that directly target components of the ECM, one example is the inhibition of FN biosynthesis. Tomasini-Johansson et al. developed a small peptide inhibitor, pUR4B, which effectively limits the polymerization of FN in the matrix [177]. pUR4B was also demonstrated by Hielscher and colleagues in co-cultures of fibroblasts and breast cancer cells to reduce not only the deposition of FN in the ECM and organization, but also to inhibit the deposition of other stromal proteins [178]. However, to date, neither strategy has been validated in studies of GI cancers.

### 5.2. Awakening Dormant Cancer Cells to Re-Sensitize Them to Therapy

Based on a growing understanding of the mechanisms of cancer dormancy, the prevention of cancer cells from entering dormancy or forcing DCCs to awaken, thereby increasing cellular sensitivity to therapeutic agents, is a novel strategy that may be developed to combat the recurrence of drug-resistant disease. The inhibition of dormancy-promoting factors influenced by CAFs may be one way to achieve this. For example, TGF-β2 binds to the TGF-βRIII signaling pathway and induces tumor cells to enter a dormant state. AP 12009 (also known as Trabedersen), an antisense oligonucleotide (ASO) targeting TGF-β2 mRNA, has been shown to be safe in phase I/II studies in patients with pancreatic and colorectal cancer [179].

CAF-secreted DKK-1 is a key factor in the promotion of cancer cell dormancy. DKN-01 is a humanized monoclonal antibody that binds to and blocks the activity of the DKK-1, and it has been evaluated in several clinical trials for GI cancers. A phase II study of DKN-01 in combination with Tislelizumab with or without chemotherapy as first line therapy for patients with metastatic gastric cancer or gastroesophageal junction adenocarcinoma is ongoing [180,181]. Another a phase Ib clinical trial showed that DKN-01 in combination with pembrolizumab was well tolerated in advanced esophageal cancer patients [182]. In addition, a phase II study of DKN-01 in combination with standard-of-care bevacizumab (Avastin) and chemotherapy as second-line treatment for patients with advanced CRC (NCT05480306) is ongoing. However, in these clinical trials, the effectiveness of the treatment may depend on the amount of DKK1 that is expressed by the patient’s own tumor.

Other factors secreted by CAFs, such as GDF-10 and BMP4, have been reported to be involved in the promotion of cancer cell dormancy. Treatment targeting these factors has not been investigated in GI cancers [113,183,184]. In the future, related antibodies, RNA interference, or inhibitors may have potential therapeutic effects and become treatment options for GI cancers. In conclusion, by inhibiting the CAF-derived factors that promote dormancy in the TME, tumor cells can be prevented from entering or awakened from dormancy. It is recommended that this be carried out early in the treatment process to prevent the tumor cells from acquiring a strong malignant capacity. Table 1 summarizes the factors secreted by CAFs that have been targeted by agents used in GI cancers. The table highlights potential strategies that could be applied to target cancer dormancy at different stages.

## 6. Conclusions

The communication between cytokines/chemokines secreted by CAFs and other components of the TME, as well as ECM remodeling and immune modulation, are involved in the development of cancer (Figure 1), which includes resistance to treatment, dormancy, and metastasis. Metastatic recurrence is a significant problem in patients with GI cancers and is a leading cause of death. Cancer dormancy was initially identified in breast cancer, where cancer cells remain inactive for a prolonged period before recurrence [185]. However, less research has been performed on dormancy associated with GI-related cancers.

This review focused on how CAF secretion affects the GI TME and its impact on cancer dormancy (Figure 2). The decision to enter or exit cancer dormancy depends on the dynamics of the TME, with CAF secretion playing a critical role. Therefore, CAFs are potential targets for therapeutic strategies in the treatment of cancer and the prevention of recurrence and metastasis. However, some CAFs have dual roles in the cancer cell dormancy/awakening mechanism [186,187]. Early in treatment, CAFs may help cancer cells enter dormancy and survive treatment, while at later stages, CAFs create a microenvironment conducive to metastasis, thus allowing cancer cells to escape from dormancy and metastasize with greater malignancy. Further studies are needed to evaluate the specific responses of each secreted factor in detail.

This review summarized several potential approaches to targeting CAF secretion, such as maintaining cancer cells in a dormant state indefinitely or awakening DCCs to re-sensitize them to treatment (Figure 3). However, these approaches have their complexities and require the consideration of the treatment window and patient stratification. Ideally, the former should be used in the post-treatment phase, and it may require a long period of continuous application. The latter should be used from the early stages of treatment and in combination with other treatments such as chemotherapy or immunotherapy [188].

Taken together, it is reasonable to believe that CAF-driven secretion in the TME can be used as a strategy for combating GI cancer recurrence, which scientifically and theoretically provides an insightful basis for the development of anti-cancer treatment.

## Figures and Tables

**Table 1:** Agent targeting CAF-secreted cytokines/chemokines that maintain or awaken cancer cell dormancy.

Function	Target	Agent	Mechanism	Clinical Trial	Ref.
Maintain Dormancy	uPAR	ATN-292	Blocking uPA/uPAR interaction to inhibit ERK signaling; preventing the reawakening of DCCs	Preclinical development	[135]
	uPA	WX-671		Phase II (NCT00499265)	[137]
	TGF-β1	SRK-181	A selective antibody against TGF-β1	Phase I (NCT04291079)	[139,141,142]
	TGF-βRI	LY2157299	Inhibitor of TGF-βRI kinase, blocking the TGF-β1-induced dormancy escape signaling pathway	Phase Ib/II (NCT02734160)	[143,144,145]
		Ki26894		Preclinical development	[146]
	COX-2	Sulindac	Non-steroidal anti-inflammatory molecules that may inhibit inflammation-mediated escape from dormancy	Phase III (NCT01349881)	[147,148,149]
		Celecoxib		Phase III (NCT01150045)	[152,153]
	α5β1	Volociximab	Inhibition of ECM remodeling involved in dormant escape	Phase Ib (NCT00654758)	[163]
		THR-687		Phase II (NCT05063734)	[164]
	β1 integrin	OS2966	Inhibition of ECM remodeling involved in dormant escape	Phase I (NCT04608812)	[165]
	LOX	BAPN	Inhibit the organization of collagen fibers; target the microenvironment that supports dormant awakening	Preclinical development	[168,169]
	LOXL2	Simtuzumab	Inhibit the organization of collagen fibers; target the microenvironment that supports dormant awakening	Phase II (NCT01472198) Phase II (NCT01479465)	[170,171]
	TGF-β1/LOXL2	EGCG	Inhibition both of TGF-β1 signaling and collagen accumulation	Preclinical development	[173]
	TSP-1	Prosaposin	Enhancing the dormant driver as well as the microenvironments	Preclinical development	[175]
	TGF-β2	5-AZA with ATRA	Restores the NR2F1-induced dormant program and induces TGF-β2, thereby inhibiting metastasis by restoring TGF-β-SMAD4 signaling	Preclinical development	[176]
Awaken Dormancy	TGF-β2	AP 12009	An antisense oligonucleotide (ASO) targeting TGF-β2 mRNA; blocking the dormancy driver	phase I/II (NCT00844064)	[179]
	DKK-1	DKN-01	A humanized antibody blocks DKK-1 activity and restores Wnt signaling and cell proliferation	phase I (NCT02013154) phase II (NCT04363801) phase II (NCT05480306)	[180,181,182]
